# Annotations

**Published:** 1901-07-06

**Authors:** 


### ANNOTATIONS.

### School Board Certificates.

At the South-western Police Court the perennial squabble about the School Board and its medical certificates came up again last Saturday. A man was summoned for neglecting to send his child to school, and on the case first being heard the mother had produced a certificate from Dr. Myles to the effect that the child was suffering from valvular disease of the heart and was unable to attend school. On the application of Mr. A. T. Burton, acting on behalf of the London School Board, the magistrate granted an adjournment to allow of the child's being examined by Dr. Kempster for the Board. The certificate of this gentleman practically coincided with that of Dr. Myles. Unfortunately, Dr. Kempster had chosen to insert in his certificate (we go by the report in the *Times*) the assertion that "the child would be just as well in school as nursing a baby at home," and Mr. Burton suggested that the magistrate should make an order for the child to attend school. Dr. Myles, however, attended as a witness, and in the end the magistrate very wisely refused to take the responsibility of making an order as suggested and adjourned the summons *sine die.* When will the School Board learn decency? and instead of trying to force admittedly sick children to go to school try to collect some of the dirty urchins who run wild about the streets. Too often, we believe, the trouble arises from the obstinacy of the School Board visitors who, instead of making reasonable inquiry prefer to make a show of their authority; but surely the officials at the Board who have the control of these matters, after asking for and making use of medical certificates for which they persistently refuse to pay a farthing, ought to have the decency to accept these certificates and to act upon them. But no, it appears to be the theory of the Board that their hold upon a district is lessened if in a single case their visitor is beaten. Hence we find the full force of the Board brought to bear to force a child with heart disease to go to school contrary to the doctor's advice. What is one child to the Board, compared with the ineffable advantage of letting all the mothers of a district know that the word of the School Board visitor is law?

### The Open Shop.

The question of the open shop has been brought prominently forward by a case in which a practitioner in Glasgow was charged before the General Medical Council as follows, namely, "that he being a registered medical practitioner habitually employed as assistants, for the sale of drugs and poisons, persons who are not qualified to act as chemists or pharmaceutical assistants, and thereby causes such persons to commit breaches of the Pharmacy Acts." That the Council muddled the matter almost goes without saying. It "considered the charge proved" but it did not judge the defendant "guilty of 'infamous conduct in a professional respect.'" Nevertheless it went on, by its President, to lecture him on his conduct in employing unqualified assistants to sell, not "poisons" as in the charge brought against him, but "scheduled poisons," things never mentioned in the charge at all, and ended by saying that it had decided to deal leniently with him. For a judicial body to play such pranks is lamentable. Clearly the Council should have insisted upon the charge being amended before it was considered. But, at any rate, the outcome of the whole affair is obvious enough, namely that, notwithstanding the fact that the Pharmacy Acts contain clauses exempting medical practitioners from their operation, this clause is not to be read as covering their assistants, and that if medical practitioners choose to keep open shop and to sell scheduled poisons to the public they must either do the work themselves or must employ assistants qualified under the Pharmacy Acts. In fact we are not quite clear that even qualified medical practitioners themselves have any right to sell scheduled poisons except when contained in medicines prescribed by them for their patients; and if that should turn out to be the case there is an end to the open shop. And a good thing too. A broad distinction must always be drawn between the dispensing of medicine by a medical practitioner—whether it is done by himself or by his assistant—and the sale of medicines (and many other things) over the counter. That medical practitioners should dispense their own medicines is in many cases greatly to the advantage of their patients and the public, however disagreeable the task may be, and in some country places it is absolutely necessary. But the keeping of an open shop is another affair. It is absolutely unnecessary, it is a most unseemly appendage to a medical man's professional work, and, as evidently has been the case in Scotland, it is liable to great abuse.

### The Medical Missionary.

From the point of view of religion, and especially of a religion which regards missionary effort as one of the most important of duties, the medical missionary is a most useful person. To preach one must get hearers. No man enters into such confidential relation with his fellows as does the doctor with his patient; no man has better opportunities of saying "a word in season," and, therefore, no man can be better able to spread the faith. And the world is now full of medical missionaries; all of which is good, and from the point of view of those who send them out is admirable. Whether it is quite in accord with professional ethics is another matter. We do not wish to dogmatise, and certainly we do not wish to speak lightly of the self-sacrificing missionary efforts which are being made on every side; still the fact remains that directly a medical man steps aside from his medical work he enters upon a dubious path which, plain as it may seem to him, is full of prickles from other points of view. Of course every man may preach his faith in the market-place and elsewhere; but the question is how far a man whose intimate relations with his patient are purely professional may use—or as those of other faiths would say may abuse—this professional relationship to whisper "words in season," or, to put it plainly, to act as a proselytising agent? Notwithstanding the many political complications which have arisen from the practice, most of us hold to our perfect right to dose the heathen with mingled physic and religion; but the story told in the House of Commons as to the doings of Dr. Long in Limerick, and the riotous proceedings which resulted from the establishment of his dispensary, showed just how the medical missionary is apt to be looked at in a civilised country by those who regard his medical ministrations as merely a cloak to attempts to undermine their religion. How, then, about the feelings of the poor heathen? We are not going to take a side in this question. We merely insist that there *is* a question, and that it is not so absolutely clear as some have thought that it is right for a medical man to use his professional position as a means of preaching his religion.

